# Evolutionary Ecology of Plant-Arthropod Interactions in Light of the “Omics” Sciences: A Broad Guide

**DOI:** 10.3389/fpls.2022.808427

**Published:** 2022-04-25

**Authors:** Ivan M. De-la-Cruz, Femke Batsleer, Dries Bonte, Carolina Diller, Timo Hytönen, Anne Muola, Sonia Osorio, David Posé, Martijn L. Vandegehuchte, Johan A. Stenberg

**Affiliations:** ^1^Department of Plant Protection Biology, Swedish University of Agricultural Sciences, Alnarp, Sweden; ^2^Terrestrial Ecology Unit, Department of Biology, Ghent University, Ghent, Belgium; ^3^Department of Agricultural Sciences, Viikki Plant Science Centre, University of Helsinki, Helsinki, Finland; ^4^NIAB EMR, West Malling, United Kingdom; ^5^Biodiversity Unit, University of Turku, Finland; ^6^Departamento de Biología Molecular y Bioquímica, Instituto de Hortofruticultura Subtropical y Mediterránea “La Mayora”, Universidad de Málaga-Consejo Superior de Investigaciones Científicas, Campus de Teatinos, Málaga, Spain; ^7^Department of Biology, Norwegian University of Science and Technology, Trondheim, Norway

**Keywords:** plant-insect interactions, natural selection, metabolomics, genomics, plant defenses

## Abstract

Aboveground plant-arthropod interactions are typically complex, involving herbivores, predators, pollinators, and various other guilds that can strongly affect plant fitness, directly or indirectly, and individually, synergistically, or antagonistically. However, little is known about how ongoing natural selection by these interacting guilds shapes the evolution of plants, i.e., how they affect the differential survival and reproduction of genotypes due to differences in phenotypes in an environment. Recent technological advances, including next-generation sequencing, metabolomics, and gene-editing technologies along with traditional experimental approaches (e.g., quantitative genetics experiments), have enabled far more comprehensive exploration of the genes and traits involved in complex ecological interactions. Connecting different levels of biological organization (genes to communities) will enhance the understanding of evolutionary interactions in complex communities, but this requires a multidisciplinary approach. Here, we review traditional and modern methods and concepts, then highlight future avenues for studying the evolution of plant-arthropod interactions (e.g., plant-herbivore-pollinator interactions). Besides promoting a fundamental understanding of plant-associated arthropod communities’ genetic background and evolution, such knowledge can also help address many current global environmental challenges.

## Introduction

In response to the damage caused by herbivorous insects, plants have evolved both direct defenses (e.g., chemical compounds and physical barriers) and indirect defenses (e.g., volatile organic compounds, VOCs) that attract—or enhance the effectiveness of—herbivores’ natural enemies (Kessler and Baldwin, 2002; Poelman et al., 2008; Mithöfer and Boland, 2012). Furthermore, defensive traits may also attract or repel pollinators (Galen and Cuba, 2001; Ramos and Schiestl, 2020; Egan et al., 2021). Defensive traits can thus affect herbivores, their natural enemies, and pollinators (Bukovinszky et al., 2008; Poelman et al., 2008). Recent evidence has shown that pollinators are also important selective agents of both plant defensive and reproductive traits (Herrera et al., 2002; Strauss and Irwin, 2004; Kessler and Halitschke, 2009; Kessler et al., 2011; Adler et al., 2012; Campbell and Kessler, 2013; Muola et al., 2017; Ramos and Schiestl, 2019, 2020; Rusman et al., 2019; Santangelo et al., 2019; Egan et al., 2021; Table 1). For instance, plants profit from large, colorful flowers that attract pollinators, but such attractive signals sometimes also attract herbivores, imposing an ecological trade-off on the signals’ evolution (Ramos and Schiestl, 2019; Egan et al., 2021). Indeed, plant-pollinator-herbivore interactions, and the mediating traits, are often interdependent and have context-dependent ecological outcomes (Kessler et al., 2011; Ramos and Schiestl, 2019, 2020; Santangelo et al., 2019; Kessler and Chautá, 2020; Egan et al., 2021; Table 1). Predators and parasitoids are also selective agents of plant defenses, especially indirect defenses (Kessler and Heil, 2011). For example, the secretion of extrafloral nectar and VOCs can attract ants or parasitic wasps that are predators and parasitoids, respectively, of herbivores (De Moraes et al., 1998; Heil et al., 2001; Kessler and Heil, 2011; Zhang et al., 2019).

**Table 1:** Key examples of publications addressing the genetic basis of adaptation, eco-metabolomics, and plant-herbivore-pollinator interactions.

General topic	Reference	Description
Herbivory, pollination, and plant defenses		
	Adler et al., 2012	Selection by pollinators shapes the evolution of floral and leaf chemical defenses.
	Herrera et al., 2002	One of the first studies suggesting correlated evolution of mutualism- and antagonism-related plant traits.
	Kessler and Halitschke, 2009	Testing the potential for conflicting selection on floral chemical traits by pollinators and herbivores.
	Muola et al., 2017	Analysis of direct and pollinator-mediated effects of herbivory.
	Ramos and Schiestl, 2019	Analysis of rapid plant evolution driven by the interaction of pollination and herbivory.
	Ramos and Schiestl, 2020	Analysis of herbivory and pollination effects on the evolution of herbivore-induced plasticity in defense and floral traits.
	Santangelo et al., 2019	Study indicating that herbivores and plant defenses affect selection on plant reproductive traits more strongly than pollinators.
Genetic basis of trophic level interactions		
	Bailey et al., 2006	Study showing that phytochemistry and genetically based species interactions are important components of community heritability and ecosystem processes.
	Whitham et al., 2006	A framework for community and ecosystem genetics: from genes to ecosystems.
	Dungey et al., 2000	Study showing how plant genetic factors affect arthropod community richness and composition.
	Gomulkiewicz et al., 2000	Modeling illustrating the geographic mosaic theory of coevolution.
	Schuman and Baldwin, 2018	Field studies revealing functions of chemical mediators in plant interactions.
	Thompson, 2005	Analysis of the geographic mosaic of coevolutionary arms races.
Genomics of adaptation		
	Anderson et al., 2014	Review highlighting the importance of field studies for advancing our understanding of evolutionary genetics.
	Brachi et al., 2011	Review addressing why GWAS in plants have been successful, focusing on the experimental designs and sampling strategies used.
	Bradshaw et al., 1998	One of the first empirical studies providing evidence of QTLs of flower morphology.
	De Mita et al., 2013	Guidelines for the use of popular or recently developed statistical methods to detect footprints of selection with genomic data.
	de Villemereuil et al., 2016	Review highlighting the use of common garden experiments in the genomic era.
	Dyer et al., 2018	Review of modern approaches to study plant–insect interactions.
	Fraser, 2020	Introduction of an approach for detecting selection with a genetic cross.
	Merilä and Crnokrak, 2001	Pioneering study on the comparison of genetic differentiation at marker loci and quantitative traits.
	Savolainen et al., 2013	Review on ecological genomics of local adaptation.
	Sork, 2018	Genomic studies of local adaptation in natural plant populations.

The eco-evolutionary roles of herbivores, their enemies, and pollinators in shaping plant traits (e.g., defenses and floral attraction) have been typically studied in isolation and under controlled conditions (but see Herrera et al., 2002; Strauss and Irwin, 2004; Kessler and Halitschke, 2009; Adler et al., 2012; Ramos and Schiestl, 2019, 2020; Santangelo et al., 2019; Egan et al., 2021; Table 1). However, multiple arthropod species within a biotic community may have significant effects on the evolution of any plant trait through diffuse coevolution (Fox, 1988; Strauss et al., 2005). Therefore, studies on the complex interactions between plants and their associated communities of arthropods and other organisms are crucial to elucidate their diverse direct and indirect effects on plants and vice versa (Stam et al., 2014). Moreover, many studies of plant-arthropod interactions do not account for plant genotype × environment interactions, so it is unclear whether changes in expression of examined traits have genetic (inherited) components or are plastic responses (Falconer, 1960). In addition, although there is a long-standing interest in the reciprocal selection aspects of plant-arthropod interactions (Ehrlich and Raven, 1964; Schoonhoven et al., 2005), a better understanding is needed of their responses to environmental changes (natural and anthropological) at both molecular and ecological levels (Visser, 2008; Hamann et al., 2021). Thus, more studies that include manipulations of important environmental variables (e.g., water and nutrient availability) are needed to elucidate their effects on plant-arthropod interactions (Hamann et al., 2021).

Advances in the omic sciences (metabolomics, genomics, transcriptomics, proteomics, and bioinformatics) and species’ natural history allow the acquisition of a deeper fundamental understanding of the evolutionary ecology of plant-arthropod interactions (Zheng and Dicke, 2008; Whiteman and Jander, 2010; Dyer et al., 2018; Wetzel and Whitehead, 2020; Li and Gaquerel, 2021). For example, they enable elucidation of the genetic architecture (numbers and genomic locations of genes that affect a trait, the magnitude of their effects, and the relative contributions of additive, dominant, and epistatic gene effects) of plant traits involved in interactions with herbivores, predators, and pollinators. Furthermore, such deeper fundamental knowledge and techniques can help address many practical problems. For instance, they can provide information about functions of genes involved in the expression of defensive and floral traits that can help crop breeders to search for beneficial allelic variants or novel traits to introgress from wild germplasm (Green et al., 2020; Stenberg and Ortiz, 2021). This is potentially important for various reasons. One is that many resistance traits and attractive traits have been lost during crops’ domestication. Direct defensive traits were often selected against as they interfered with crops’ taste and texture (Moreira et al., 2018), while attractive traits were rarely consciously selected against but were ignored due to lack of awareness of their importance (Green et al., 2020; Stenberg and Ortiz, 2021). Due to such complications, a multidisciplinary approach is required to enhance understanding of how natural selection mediated by plant-arthropod interactions shapes plant populations’ genetic and chemical (secondary metabolites) composition.

This mini-review focuses on traditional and modern methods and concepts, and then highlights future avenues for studying the evolution of plant-arthropod interactions, particularly those occurring above ground. First, we discuss traditional experimental designs to study the evolution of plant-arthropod interactions. Next, we address the integration of traditional experiments with new technologies to study plant-arthropod interactions, eco-metabolomic approaches to explore plant-arthropod interactions, and integration of quantitative genetic/genomic analyses with metabolomic techniques. Finally, we present conclusions and opportunities for deeper elucidation of plant-arthropod interactions in the genomics era in light of climate change. In summary, this mini-review provides general information on approaches for studying plant-arthropod interactions at molecular and ecological levels. Besides enhancing fundamental understanding of the evolution of plant-arthropod interactions in ecosystems, such information is essential for tackling major current global environmental challenges.

## Common Garden and Reciprocal Transplant Experiments to Study Local Adaptation of Plant-Arthropod Interactions

**Figure 1:**
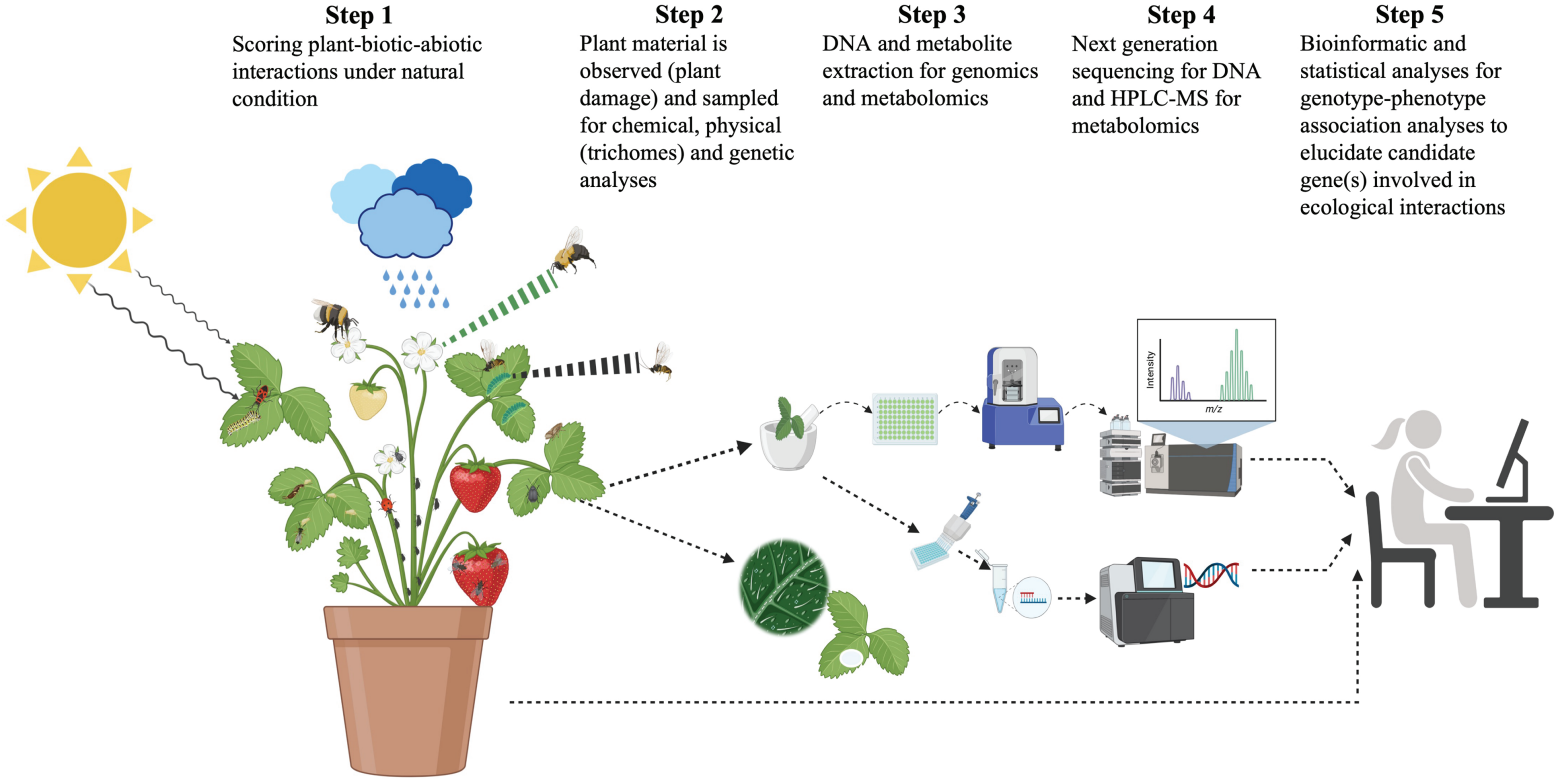
Schematic diagram of steps in the study of plant-herbivore-pollinator interactions. Plants are attacked by various herbivore species and pollinated by different species at the same time in changing environments. They produce direct (e.g., alkaloids, trichomes, and spines) and indirect defenses [volatile organic compounds (VOC) and green leaf volatiles] that provide varying degrees of protection to their natural enemies and at the same time, these secondary compounds can attract pollinators. Direct defenses have a direct negative effect on the enemies’ fitness and/or performance, whereas indirect defenses attract predators and parasitoids of herbivores. Defenses can be induced (produced after damage) or constitutive (produced all the time). The production and accumulation of chemical defenses in leaves and flowers directly affect the attraction of pollinators. Pollinators can hence also mediate plants’ chemical defenses and plant-herbivore interactions. Defensive traits and herbivores, predators/parasitoids, and pollinators are measured in natural conditions, and leaf and flower tissues are collected for metabolomics (HPLC- and/or GC–MS-based) and genomic analyses to identify genotype–phenotype associations [quantitative trait loci (QTL) analyses, genome-wide association analysis (GWAS), and/or identity-by-descent analyses] and thus loci mediating plant-herbivore-pollinator interactions.

Intraspecific genetic variation in plant populations has consequences for associated communities of arthropods and other organisms (Dungey et al., 2000; Hochwender and Fritz, 2004; Johnson and Agrawal, 2005; Wimp et al., 2005; Bailey et al., 2006; Vandegehuchte et al., 2011; Moreira and Mooney, 2013; Koricheva and Hayes, 2018). Indeed, plant genotypic variation may have even stronger effects than environmental factors on arthropod communities’ composition and interactions (Johnson and Agrawal, 2005; Bailey et al., 2006; Koricheva and Hayes, 2018). Furthermore, spatial variation in environmental characteristics across populations may result in a selection mosaic that favors different traits (or trait values) in different locations, thereby promoting phenotypic and genetic/genomic divergence among populations in plant traits. Examples include adaptation to local insect communities (Gomulkiewicz et al., 2000; Thompson and Cunningham, 2002; Thompson, 2005). Thus, a key issue for evolutionary ecologists studying plant-arthropod interactions is how plants adapt to and cope with antagonistic and mutualist insects simultaneously (Figure 1).

**Figure 2:**
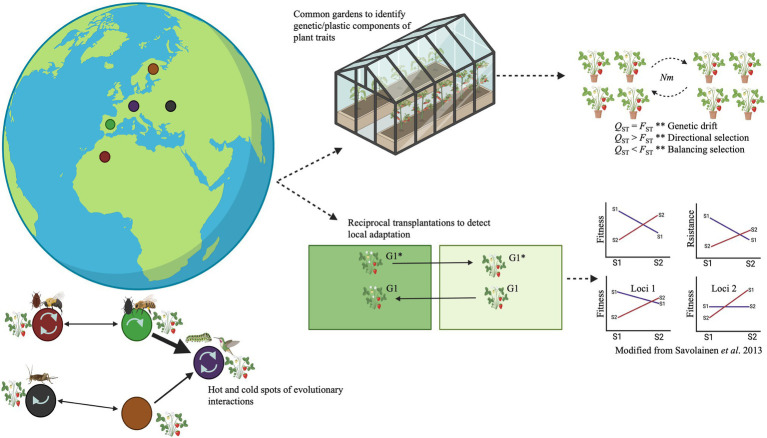
The geographic mosaic of coevolution theory holds that interacting species sometimes impose reciprocal natural selection pressures on each other (coevolution). It shapes interactions between pairs of species, small groups of species, and large webs of species. Species are often collections of genetically distinct populations, interacting species often differ in their geographic ranges, and interactions among species differ among environments in their ecological outcomes (colored circles). The colored circles represent biological communities or populations; the arrows in them indicate interactions within local communities and selection on the plant by one, more than one (in hotspots), or none of the associated species (in cold spots). The arrows between communities indicate gene flow (thicker arrows = higher gene flow). Genotype–phenotype association analyses (e.g., QTL analysis, GWAS, and/or identity-by-descent analysis) allow to study of the geographic mosaic of coevolution at the genomic level. Traditional experimental approaches such as common garden and reciprocal transplant experiments have been used to study local adaptation at the ecological level. In common gardens, plants from different populations are grown in the same environment to assess whether phenotypes of interest have a genetic component. In contrast, reciprocal transplant experiments enable comparisons of the relative fitness of a population in its native environment and another environment (home vs. away comparison), and the fitness of native and foreign populations in the same environment (local vs. foreign comparison). Genotypes G1 and G1* are planted in their local and foreign environments. Genomic, metabolomic, and bioinformatic analyses allow detection of local adaptation of interactions at the genomic level *via* genotype–phenotype association analyses or *Q*_ST_ vs. *F*_ST._

To study local adaptation of plant defensive traits to local insect communities, it is necessary, in general terms, to elucidate whether a population has higher fitness at its native site than any other population introduced to that site (Kawecki and Ebert, 2004; Blanquart et al., 2013; Savolainen et al., 2013; Figure 2). From a genomic perspective, local adaptation should occur when a population has a genome-wide complement of available alleles that maximizes fitness in the local environment (Savolainen et al., 2013; Figure 2). Phenotypic and genetic differentiation along environmental gradients, or across contrasting habitat types, can also indicate local adaptation (Merilä and Crnokrak, 2001; Leinonen et al., 2013; Savolainen et al., 2013; Anderson et al., 2014; Figure 2). Evolutionary biologists have used traditional approaches such as common garden and reciprocal transplant experiments to detect local adaptation and the genetic architecture of complex traits (e.g., plant defenses; Figure 2). However, something to remark is that a single common garden experiment does not provide direct evidence of local adaptation (de Villemereuil et al., 2016). Typically, just one common garden is used. In such cases, complementary methods such as the use of molecular markers (e.g., single-nucleotide polymorphisms/SNPs and microsatellites) must be used together with a common garden experiment to detect evidence of local adaptation (see Merilä and Crnokrak, 2001; Savolainen et al., 2013; de Villemereuil et al., 2016; De-la-Cruz et al., 2020a). Common garden and reciprocal transplant experiments allow the exclusion of the pervasive confounding effects of other evolutionary phenomena such as genetic drift, phenotypic plasticity, complex demographic history, and complex genetic architecture (Anderson et al., 2014; de Villemereuil et al., 2016). Reciprocal transplant experiments enable comparison of populations’ fitness in their native and other environments (i.e., home vs. away comparison), and in environments that are native to one but foreign to other populations (i.e., local vs. foreign comparison; Kawecki and Ebert, 2004; Ågren and Schemske, 2012; Anderson et al., 2014; Figure 2). In practice, reciprocal transplantations provide direct evidence of local adaptation if populations have higher fitness (e.g., higher survival or reproductive success) under “home” and “local” treatments than under “away” and “foreign” treatments (Kawecki and Ebert, 2004; Ågren and Schemske, 2012; Savolainen et al., 2013; Anderson et al., 2014; Figure 2). A complication is that fitness measurements in many plant species are hard to assess. For example, plants that produce more seeds are often assumed to have greater fitness than conspecifics that produce fewer seeds (Primack and Kang, 1989). Nevertheless, plants that produce relatively few seeds may have higher quality than plants that produce many seeds, so the plants with low fecundity may actually leave more offspring in future generations (Primack and Kang, 1989). Therefore, the use of common garden experiments together with genetics tools (see below) may be valuable alternatives since they enable measurements of local adaptation that do not completely depend on fitness measurements (Merilä and Crnokrak, 2001; Savolainen et al., 2013; de Villemereuil et al., 2016; Figure 2). Furthermore, common gardens allow controlling for the effects of phenotypic plasticity by growing individuals from different populations in a common environment (Leinonen et al., 2013; Savolainen et al., 2013; de Villemereuil et al., 2016; Figure 2). Common garden experiments can also be used to study responses of plant genotypes (genotype-by-environment interactions) to different insect communities by implementing the same design in different environments (de Villemereuil et al., 2016; Figure 2). Indeed, this can be considered as an extension of reciprocal transplant experiments if fitness data are collected in multiple common gardens (de Villemereuil et al., 2016). Replication of the experiments (e.g., multi-year studies) is recommended depending on whether *a priori* knowledge or a hypothesis exists about environmental factors relevant for the divergent selection that drives local adaptation (Kawecki and Ebert, 2004).

## Integrating Traditional Experiments With New Technologies to Study Plant-Arthropod Interactions

The advent of whole-genome sequencing has allowed the exploration of genotype–phenotype associations at the genomic level. Nowadays, quantitative trait loci (QTL) analyses, association mapping approaches, transcriptomics, metabolomics (gas and liquid chromatography coupled with mass spectrometry, GC/LC–MS), and populations genomics, along with reciprocal transplant experiments, can be used to elucidate the plant genetic and metabolic bases of adaptations on local arthropod communities (Zheng and Dicke, 2008; Whiteman and Jander, 2010; Savolainen et al., 2013; de Villemereuil et al., 2016; Dyer et al., 2018; Sork, 2018; Wetzel and Whitehead, 2020; Li and Gaquerel, 2021; Figures 1, 2). For instance, common gardens can be established along gradients differing in natural conditions (e.g., latitude and/or altitude; Hahn et al., 2019; Galmán et al., 2021; López-Goldar and Agrawal, 2021). Each common garden must be populated with the same genotypes and replicates (full-sibs, half-sibs, and clones) to allow partitioning of the genetic variation within and between populations and to elucidate phenotypic plasticity (Falconer, 1960; Lynch and Walsh, 1998). One of the advantages of exposing plants/genotypes to natural environmental conditions is that natural selection can be tested in the context of the natural history of the complex interactions between plants and arthropods (Schuman and Baldwin, 2018). To this end, seeds of multiple plant genotypes are sometimes germinated under controlled conditions (temperature, watering, and light) to account for environmental effects on germination and seedling survival, then transferred as seedlings to natural environments. Ideally, if genotypes are germinated for several generations under controlled conditions, it is also possible to rule out the possible maternal effects (any initial differences in plant performance due to maternal effects on seeds should have disappeared). Several traits related to plant-arthropod interactions (e.g., herbivore-predator-pollinator interactions) can be assessed, e.g., by recording numbers and identities of herbivores (e.g., folivores, florivores, and fruit predators), predators, parasitoids, and pollinators observed on monitored plants (Stam et al., 2014; Turlings and Erb, 2018; Figure 1).

A feasible technique for counting arthropods that reside on or visit a plant is randomly choosing a certain number of branches, leaves, or other appropriate organs. For example, to record pollination rates, the most common and straightforward approach is to observe pollinator visits on a set of flowers during a certain period of time. This gives an idea of the diversity of pollinators and their visitation frequencies. An alternative is the use of radars, such as telemetry and electronic tagging, that combined with cameras allow tracking individual pollinators and flower visitations (Hagen et al., 2011; Abdel-Raziq et al., 2021; Smith et al., 2021; Sun and Gaydecki, 2021). Likewise, quantum dots (semiconductor nanocrystals) can be used as pollen-grain labels to study the fate of the pollen (Minnaar and Anderson, 2019). Other more elaborate methods, such as evaluating the relative success of single visits in terms of fruit and seed set, can provide further information on visiting pollinators’ pollination effectiveness (i.e., quality of pollen transfer).

On the other hand, counting parasitoid visitation frequencies is complicated by their small size and mobility (they usually only spend a few seconds on plants). A direct method to identify parasitoids is the use of trap nests (Staab et al., 2018). However, trap nests are not optimal for studies aiming to detect the ongoing natural role of the parasitoids on their prey abundance (plant-herbivore-parasitoid interaction). For instance, if the parasitoids are caught, their population sizes will be artificially altered, which could affect the herbivore community’s performance. An indirect method is to count relative numbers of mummified prey (e.g., aphid and larva mummies) on the plants.

Predation rates can be estimated using various methods; e.g., plasticine dummy larvae can be cheap and convenient tools to assess predation rates (Howe et al., 2009; Low et al., 2014; Sam et al., 2015; Hertzog et al., 2017; Roslin et al., 2017). However, if they are used it is important to consider the size, material, and color of the dummy larvae as some predators are more chemically or visually oriented than others (Low et al., 2014). An advantage of plasticine larvae is that they enable recognition, at least at group taxonomy level, of predators attacking the larvae by identifying predation marks (Low et al., 2014). Placing live aphids or caterpillars on plants is an alternative to estimate predation rates on them (Schuman et al., 2012; Lövei and Ferrante, 2017).

Although direct and indirect traditional approaches have been used to characterize (e.g., diversity, abundance, and interactions) the arthropod community on plants, these observations are typically time-intensive, limited by environmental conditions and logistics, and they are not conducted over large spatiotemporal scales and may underestimate the importance of mobile and small organisms compared to larger or slower ones (Micheneau et al., 2010; Droissart et al., 2021). Moreover, another uncertainty associated with observational methods is taxonomic identification (i.e., classification of many insect herbivores to species level; Derocles et al., 2018; Zhu et al., 2019). Thus, alternative methods based on sensor-based insect monitoring, such as camera trap technology, can greatly improve studies of plant-arthropod interactions by providing a convenient replacement or complement to classic human observations (Minnaar and Anderson, 2019; Droissart et al., 2021). Such methods are particularly well suited for the study of pollination, insect behavior, and predator–prey interactions (Droissart et al., 2021; Høye et al., 2021). Sensor-based insect monitoring allows obtaining hundreds of pictures, videos, and recordings for deep learning analyses such as validation-image-based taxonomic identification to obtain database references of the local arthropod community interacting with the plants (Høye et al., 2021). Likewise, molecular methods such as DNA barcoding and metabarcoding techniques are increasingly applied to food web studies with the development of sequencing techniques (García-Robledo et al., 2013; Wirta et al., 2014; Kartzinel et al., 2015; Baker et al., 2016; Pornon et al., 2016; Kress, 2017; Zhu et al., 2019) and contribute to solving the problem of low species resolution and diet identification efficiency. For instance, animal DNA barcoding of COI (mitochondrial cytochrome c oxidase 1) has been widely used to identify parasitoids for constructing feeding associations between hosts and parasitoids (Wirta et al., 2014; Derocles et al., 2018; Zhu et al., 2019).

Herbivore damage is also an important trait to consider in investigations of plant-arthropod interactions (Johnson et al., 2016; Stenberg and Muola, 2017). Various non-destructive methods can be used to estimate herbivore damage, such as field observations of each damaged leaf (Johnson et al., 2016; Stenberg and Muola, 2017; De-la-Cruz et al., 2020b). However, for this task, observers must be trained in damage detection and estimation to minimize inter-observer bias (unless a single observer is measuring all plants). Another possibility is to use new smartphone applications that permit estimation of *in situ* plant damage by herbivores through examination of a certain number of randomly selected leaves (Johnson et al., 2016; Machado et al., 2016; Getman-Pickering et al., 2020). When leaves can be collected (usually at the end of an experiment unless a representative number of leaves can be cut during the experiment), they can be scanned or photographed and computer applications (e.g., WinFolia or PLIMAN) can be used to estimate leaf damage. The advantage of this approach over other methods is that it can provide more accurate damage scoring.

To integrate traditional experiments with new technologies to study plant-arthropod interactions it is necessary to bridge semantic gaps between evolutionary ecologists and geneticists, who often do not speak the same scientific language. For example, evolutionary ecologists refer to natural selection as an ongoing process leading to evolution (the study of natural selection in the wild), while geneticists typically focus on traces of selection in genomes. Hence, the traditional tools of the quantitative geneticist are still relevant and complementary in an integrative approach for detecting selection mediated by plant-arthropod interactions at phenotypic and genomic levels (Figures 1, 2).

## Eco-Metabolomics of Plant-Arthropod Interactions

Metabolomic analysis of plant-arthropod interactions starts with the collection of plant tissues (e.g., leaves and flowers) from plants that have been exposed to multitrophic interactions with natural arthropod communities (Dyer et al., 2018; Figure 1). Depending on the issues addressed, investigators may be interested in the variation of the so-called secondary compounds (concentration and diversity) during plants’ development and thus may need to collect relevant tissues throughout their life cycles. Likewise, investigators could be interested in the variation of secondary compounds across plant tissues (e.g., leaves vs. flowers) and their roles as defenses/attractants to herbivores, pollinators, and parasitoids. Indeed, it has been shown that metabolites from leaves can also function as attractants for pollinators and those metabolites from flowers can also attract herbivores (Kessler and Halitschke, 2009; Ramos and Schiestl, 2019, 2020; Egan et al., 2021). To this end, we recommend collecting leaves and flowers during the flowering period because plants tend to increase the allocation of nutrient resources to defenses at the flowering stage to ensure their fitness. Depending on the experimental design, more than three replicates per genotype are typically needed, and the focal plant tissue (e.g., leaves and/or flowers) should be sampled per plant (Maier et al., 2010). Ideally, plant tissues should be randomly collected across all genotypes and replicates (Maier et al., 2010). Once leaves and/or flowers per plant/genotype have been collected, they should be immediately frozen in liquid nitrogen to stop metabolic activity (Maier et al., 2010). However, storage in dry ice or solvents might be an alternative (but see Maier et al., 2010). The tissues are usually kept at −80°C until metabolites are extracted.

Gas and/or liquid chromatography coupled with mass spectrometry are the most widely used analytical techniques for profiling complex mixtures of metabolites. Essentially, there are two types of secondary metabolite profiling, targeted and untargeted (Dyer et al., 2018; Li and Gaquerel, 2021). Target metabolites and their abundance in each sample are identified *via* their retention times, *m/z* mass-to-charge ratios, compound chemical structures, and fragmentation patterns (Dyer e al., 2018; Peters et al., 2018; Li and Gaquerel, 2021). On the other hand, bioinformatic pipelines are commonly used for automated processing of the complex, multidimensional high-resolution mass spectra acquired for untargeted metabolite detection. This involves mass feature detection, alignment among samples, MS spectral deconvolution, feature normalization, missing value imputation, and multilevel statistical analyses, as reviewed by Li and Gaquerel (2021). Despite major advances in omic sciences, metabolomic aspects are still major bottlenecks because of the high diversity of secondary plant compounds and unresolved biosynthetic pathways (Peters et al., 2018; Walker et al., 2022). However, the development of powerful analytical tools based on combinations of high-resolution MS and increasingly advanced bioinformatic tools is raising capacities to acquire and translate metabolomic information into usable data to merge with other forms of omic information (Peters et al., 2018; Walker et al., 2022). Moreover, statistical descriptors from information-theoretical frameworks have been transposed to score indices of diversity and specialization from metabolome profiles, thereby allowing quantification of the reprogramming of metabolome diversity according to ecological interactions (Li et al., 2020; Li and Gaquerel, 2021; Walker et al., 2022).

## Studying Local Adaptation of Plant-Arthropod Interactions Using Quantitative Genetics/Genomics

Current research on the ecology of plant-arthropod interactions is strongly influenced by recent advances in molecular biology. In particular, the rapidly dropping price of DNA sequencing along with common garden or reciprocal transplant experiments provide an unprecedented opportunity to study the genetic basis of plant-arthropod interactions (Whiteman and Jander, 2010; Vertacnik and Linnen, 2017; Figure 1). To this end, relevant tissues can be obtained for simultaneous DNA/RNA and metabolomic analyses (Figure 1). The complete DNA sequencing of numerous individuals is already feasible for plant species with relatively small genomes (Sims et al., 2014). Using particular genomic libraries such as restriction site-associated DNA sequencing/genotyping-by-sequencing (Rad-seq/GBS) to sequence a targeted or untargeted fraction of a genome or Illumina custom libraries for whole-genome resequencing allows to obtain thousands to millions of molecular markers (single-nucleotide polymorphisms; SNPs). These molecular markers can then be used for genotype–phenotype association analyses (e.g., QTL analysis and genome-wide association analysis or GWAS) in plant-arthropod interactions (Whiteman and Jander, 2010; Kloth et al., 2012; De Mita et al., 2013; Sims et al., 2014; Goodwin et al., 2016; Flood and Hancock, 2017; Talbot et al., 2017; Vertacnik and Linnen, 2017).

### Quantitative Genetic vs. Neutral Genetic Differentiation

Quantitative genetic vs. neutral genetic differentiation (*Q*_ST_ vs. *F*_ST_) comparison is a powerful tool to study local adaptation of plant defensive traits to local arthropod communities while ruling out the effects of genetic drift (Merilä and Crnokrak, 2001; Leinonen et al., 2013; de Villemereuil et al., 2016; De-la-Cruz et al., 2020a). *Q*_ST_ vs. *F*_ST_ comparisons were first designated for neutral microsatellite markers, but nowadays, with genomics advances, it is possible to obtain SNPs for *F*_ST_ calculation (Merilä and Crnokrak, 2001; Leinonen et al., 2013; Li et al., 2019). To obtain thousands of SNPs, DNA sequences should be first filtered, aligned to a high-quality reference genome, and then the variants should be “called” (Hansen, 2016). The *Q*_ST_ vs. *F*_ST_ approach involves comparison of observed differentiation between populations in quantitative characters (e.g., *Q*_ST_ of defensive traits) with estimates of differentiation of adaptively neutral loci (*F*_ST_; Spitze, 1993; Schluter, 2000; Merilä and Crnokrak, 2001; Leinonen et al., 2013). Of three possible outcomes (*Q*_ST_ = *F*_ST_, *Q*_ST_ < *F*_ST_, *Q*_ST_ > *F*_ST_), higher differentiation in quantitative traits than in neutral loci (*Q*_ST_ > *F*_ST_) implies that directional selection is favoring different defensive phenotypes in different populations. This will probably be due to differences in arthropod communities associated with the populations (De-la-Cruz et al., 2020a,b). A family/breeding design (genotypes) is needed to obtain *Q_ST_* measurements (Spitze, 1993; Merilä and Crnokrak, 2001). However, an alternative is to use *P*_ST_, a measure of divergence that is comparable to *Q*_ST_ but based on total phenotypic variance with no distinction between the relative contribution of genetic and environmental variations (Leinonen et al., 2006, 2013). Use of *P*_ST_ instead of *Q*_ST_ is justified when estimates of additive genetic variance are not available (Leinonen et al., 2006, 2013; Lehtonen et al., 2009).

### Quantitative Trait Loci Analysis

Quantitative trait loci-mapping allows to elucidate genomic regions responsible for observed variation in quantitative traits (Bradshaw et al., 1998; Mauricio, 2001; Weinig et al., 2003; Slate, 2005; Fraser, 2020). In order to begin a QTL analysis, there are two requirements. First, there must be two or more accessions/genotypes with highly differentiated phenotypes that differ genetically with respect to the trait of interest (Mauricio, 2001; Slate, 2005; Fraser, 2020). For example, a plant genotype with high constitutive alkaloid concentrations and small flowers, and another with low constitutive alkaloid concentrations but larger flowers could be used. Parental plants of each of these genotypes must be outcrossed to obtain F_1_ progeny. The F_1_ progeny are typically self-fertilized to obtain F_2_ progeny, which can be further self-pollinated for several rounds to obtain recombinant inbred lines (RILs) that enable observation of the phenotypic/genetic segregation from the grandparents (founders). However, there are some disadvantages for bi-parental populations such as the lack of mapping precision (limited effective recombination could occur during population development) and low genetic diversity due to the genetic bottleneck caused by choice of two founders (Scott et al., 2020). Alternatively, other popular population designs for QTL analysis in plants can be used, namely, nested association mapping (NAM) and multi-parent advanced generation inter-cross (MAGIC) populations (Huang et al., 2015; Scott et al., 2020). The NAM design consists of a series of bi-parental crosses against a common founder, from which RILs are typically generated through selfing (Scott et al., 2020; Gireesh et al., 2021). In the MAGIC design, a series of equally balanced crosses are made between founders before RILs are developed (Huang et al., 2015; Scott et al., 2020).

Once phenotypes and genotypes of a derived (e.g., F_2_, MAGIC) population have been scored, molecular markers linked to a QTL influencing the trait(s) of interest will segregate more frequently with trait values (e.g., high or low alkaloid concentration and flower size), whereas unlinked markers will not be significantly associated with the phenotype (Mauricio, 2001; Slate, 2005; Fraser, 2020). Since alkaloid production and flower size could be associated with herbivore-predator-pollinator abundances, it is possible to identify the genes/alleles that affect the traits of interest (alkaloid concentration and flower size) and how they are affected by the abundance of herbivores, predators, and pollinators. Likewise, if the F_2_/MAGIC progenies are distributed in a reciprocal transplant design (in the native sites of the grandparents or two different populations), the distribution of QTL effects in the natural environments can be elucidated, and hence the genetic basis of local adaptation in plant-arthropod interactions (Mauricio, 2001; Weinig et al., 2003; Slate, 2005; Vertacnik and Linnen, 2017). The logic behind this is that the selective agents (e.g., herbivores, predators, and pollinators) should shape a specific genetic architecture in the native site of the grandparents; i.e., different alleles affecting the variance of the plant phenotypes in each native site.

### Genome-Wide Association Studies

Quantitative trait loci studies are frequently challenging since the experimental design to obtain the progenies (RILs, F2, and MAGIC) is time-consuming and requires significant work effort. Likewise, breeding can be difficult due to genetic incompatibilities between plant founders. Thus, a genome-wide association study (GWAS) provides an alternative way to overcome the challenges of a QTL study, although the genetic structure of the plant populations has to be taken into account (Brachi et al., 2011; Korte and Farlow, 2013; Talbot et al., 2017). Association analysis is based on linkage disequilibrium (LD; Brachi et al., 2011; Talbot et al., 2017) and generally involves five steps: choice of germplasm/populations, trait evaluation, population genotyping, estimation of population structure, and tests of associations between the genotypes and phenotypes (Zhu et al., 2008; Myles et al., 2009). GWAS is a powerful approach for detecting genetic variation underlying many important and complex phenotypic characters in plants, such as defensive and floral traits (see Kliebenstein et al., 2001; Aranzana et al., 2005; Huang et al., 2012; Züst et al., 2012; Katz et al., 2021). Indeed, it is possible to correlate frequencies of the alleles associated with the defensive and flower traits and their interaction with herbivore-predator-pollinator abundances across different populations. The basic adaptive premise is simple: if a single SNP (for example, an A to G variant) has low frequency in one population but high frequency in another, it may contribute to adaptation in the local environment of one or both populations (Aguirre-Liguori et al., 2021). Nevertheless, several loci/alleles frequently interact in the expression of a trait involved in local adaptation among populations (epistasis; Aguirre-Liguori et al., 2021). Evidence of local adaptation is strengthened when observed differences in allele frequencies between populations exceed expectations based on genetic drift and/or they are differentially correlated with phenotypic traits, such as chemical or physical defenses across populations (Aguirre-Liguori et al., 2021). An important advantage of GWAS over bottom-up approaches (e.g., gene silencing) is its ability to detect polygenic effects on single traits of interest, which are common as genes have interactive effects with other genes and the environment to generate phenotypes (Gibson, 2018). Other methods to detect genes under selection, and hence local adaptation, are based on *F*_ST_ outliers, site frequency spectra, and linkage disequilibrium tests (Pavlidis et al., 2008; De Mita et al., 2013; Vitti et al., 2013; Pavlidis and Alachiotis, 2017).

### Transcriptomics

Transcriptomic analyses can be used to detect differential gene expression associated with plant-arthropod interactions (e.g., plant-herbivore-pollinator interactions). For instance, treatments such as herbivory, pollination, herbivory + pollination, herbivory + predator/parasitoids + pollination treatments (Ramos and Schiestl, 2019, 2020; Pashalidou et al., 2020; Egan et al., 2021) may have illuminating effects. These may include differences in RNA expression between tissues and/or organs of interest (Koenig et al., 2013; Hekman et al., 2015), as well as during the tissues’ and organs’ development (Hradilová et al., 2017). Since transcriptomic analyses are experimental by nature, experimental designs should include biological replicates for each treatment or tissue/organ to assess the variability in the data, as well as controlled environmental conditions to reduce possible bias and sources of error (Fang and Cui, 2011; Schurch et al., 2016; Barrera-Redondo et al., 2020). Use of at least six biological replicates exposed to each condition applied in an experiment is recommended, although three replicates are commonly used (Schurch et al., 2016; Barrera-Redondo et al., 2020).

## Conclusion and Future Directions

Despite the powerful molecular, chemical, genomic, transcriptomic, metabolomic, and bioinformatic tools currently available, it is still an extremely demanding task to obtain a complete picture of the effects of plant-arthropod interactions on the evolution of plant traits at the genomic level. Furthermore, there is still a lack of empirical evidence about how plants and their herbivores and pollinators interact, and how natural selection shapes these interactions (the geographic mosaic of coevolution; Thompson, 2005). Indeed, individual plant-arthropod interactions are often studied in isolation from their ecological context. Given that local adaptation is the outcome of a dynamic balance between selection and migration, planning an experiment that investigates local adaptation of plant traits to the plant’s associated insect community always involves difficult choices and trade-offs (number of different study sites, number of screened individuals/genotypes and their replicates, and number of sequenced individuals/genotypes for population genomics), especially if it combines phenotypes with genomic data across different populations (Savolainen et al., 2013). Difficulties in sampling and monitoring multiple populations simultaneously raise further obstacles in studies of the geographic mosaic of coevolution in plant-arthropod interactions. Hence, establishing logistically feasible procedures that enable application of standardized protocols in all studied populations is essential. We believe that multidisciplinary collaboration covering all focal aspects of complex plant-arthropod interactions, at both ecological and genetic levels, is the most practical approach for this.

On the other hand, many well-characterized mutants and transgenic lines are now available for several model and non-model species (Zheng and Dicke, 2008). For instance, CRISPR/Cas9 mutagenesis, and gene silencing by RNA interference or virus-induced gene silencing have allowed the construction of specific lines of diverse species to investigate effects of individual genes (e.g., genes involved in production of specific defensive metabolites or VOCs) on individual plant-arthropod interactions in natural conditions (Zheng and Dicke, 2008; Schuman and Baldwin, 2018). However, given the long history of the interest in the reciprocal aspects of plant-arthropod interactions (Ehrlich and Raven, 1964; Schoonhoven et al., 2005), there is a need to enter the field of how non-model plant species and their associated arthropod communities respond to environmental change at the genomic, evolutionary, and ecological level. Indeed, the challenge faced by evolutionary studies of adaptation to environmental change is the difficulty of obtaining genetic evidence to differentiate between local adaptation and phenotypic plasticity as causes of observed phenotypic changes in various plant populations (Savolainen et al., 2013). Combinations of long-term field experiments with genomic analyses will enable examination of genetic changes that have occurred and estimation of strengths of selection pressures (Fournier-Level et al., 2011; Savolainen et al., 2013). Furthermore, a fundamental question is whether species will be able to adapt fast enough to track rapid environmental change (Visser, 2008; Hamann et al., 2021). Thus, more studies that include environmental manipulations (e.g., of water availability) will help to disentangle direct and plant-mediated effects of climatic factors on plant-arthropod interactions (Hamann et al., 2021). We suggest that the most realistic results would come from exposing plants together with their herbivores, predators/parasitoids, and pollinators to changing environmental conditions.

## Author Contributions

ID-l-C, AM, and JS contributed to the conception and design of the study. ID-I-C performed the literature review with the contributions of all authors and wrote the first draft of the manuscript. ID-l-C, SO, FB, DB, CD, TH, AM, DP, MV, and JS wrote sections of the manuscript. All authors contributed to the article and approved the submitted version.

## Funding

The project is funded by the European Commission as well as the following national/regional bodies: Formas—the Swedish Research Council for Sustainable Development (grant no: 2020–02376), Academy of Finland (grant no. 344726), Research Foundation—Flanders (grant no. FWO ERANET G0H6520N), and Agencia Estatal de Investigación (grant no. PCI2020-120719-2).

## Conflict of Interest

The authors declare that the research was conducted in the absence of any commercial or financial relationships that could be construed as a potential conflict of interest.

## Publisher’s Note

All claims expressed in this article are solely those of the authors and do not necessarily represent those of their affiliated organizations, or those of the publisher, the editors and the reviewers. Any product that may be evaluated in this article, or claim that may be made by its manufacturer, is not guaranteed or endorsed by the publisher.
